# 88 Lessons Learned Evaluating Ablative Fractional CO2 Laser for Burn-Related Donor Site Scars

**DOI:** 10.1093/jbcr/irae036.087

**Published:** 2024-04-17

**Authors:** Cameron S D'Orio, Bonnie C Carney, Angela Golding, Melissa M McLawhorn, Rebekah R Allely, Jeffrey W Shupp, Shawn Tejiram, Taryn E Travis

**Affiliations:** MedStar Health Research Institute, Washington, District of Columbia; MedStar Health Research Institute, Washington, District of Columbia; Medstar Washington Hospital Center, Washington, District of Columbia; MedStar Health |Georgetown University School of Medicine, Washington, DC, DC; Georgetown University School of Medicine, Washington, DC; MedStar Washington Hospital Center, Georgetown University School of Medicine, Washington, DC; MedStar Health Research Institute, Washington, District of Columbia; MedStar Health Research Institute, Washington, District of Columbia; Medstar Washington Hospital Center, Washington, District of Columbia; MedStar Health |Georgetown University School of Medicine, Washington, DC, DC; Georgetown University School of Medicine, Washington, DC; MedStar Washington Hospital Center, Georgetown University School of Medicine, Washington, DC; MedStar Health Research Institute, Washington, District of Columbia; MedStar Health Research Institute, Washington, District of Columbia; Medstar Washington Hospital Center, Washington, District of Columbia; MedStar Health |Georgetown University School of Medicine, Washington, DC, DC; Georgetown University School of Medicine, Washington, DC; MedStar Washington Hospital Center, Georgetown University School of Medicine, Washington, DC; MedStar Health Research Institute, Washington, District of Columbia; MedStar Health Research Institute, Washington, District of Columbia; Medstar Washington Hospital Center, Washington, District of Columbia; MedStar Health |Georgetown University School of Medicine, Washington, DC, DC; Georgetown University School of Medicine, Washington, DC; MedStar Washington Hospital Center, Georgetown University School of Medicine, Washington, DC; MedStar Health Research Institute, Washington, District of Columbia; MedStar Health Research Institute, Washington, District of Columbia; Medstar Washington Hospital Center, Washington, District of Columbia; MedStar Health |Georgetown University School of Medicine, Washington, DC, DC; Georgetown University School of Medicine, Washington, DC; MedStar Washington Hospital Center, Georgetown University School of Medicine, Washington, DC; MedStar Health Research Institute, Washington, District of Columbia; MedStar Health Research Institute, Washington, District of Columbia; Medstar Washington Hospital Center, Washington, District of Columbia; MedStar Health |Georgetown University School of Medicine, Washington, DC, DC; Georgetown University School of Medicine, Washington, DC; MedStar Washington Hospital Center, Georgetown University School of Medicine, Washington, DC; MedStar Health Research Institute, Washington, District of Columbia; MedStar Health Research Institute, Washington, District of Columbia; Medstar Washington Hospital Center, Washington, District of Columbia; MedStar Health |Georgetown University School of Medicine, Washington, DC, DC; Georgetown University School of Medicine, Washington, DC; MedStar Washington Hospital Center, Georgetown University School of Medicine, Washington, DC; MedStar Health Research Institute, Washington, District of Columbia; MedStar Health Research Institute, Washington, District of Columbia; Medstar Washington Hospital Center, Washington, District of Columbia; MedStar Health |Georgetown University School of Medicine, Washington, DC, DC; Georgetown University School of Medicine, Washington, DC; MedStar Washington Hospital Center, Georgetown University School of Medicine, Washington, DC

## Abstract

**Introduction:**

Hypertrophic scar (HTS) remains a comorbidity of burn injury, often requiring split thickness skin grafting (STSG) and resulting in symptomatic HTS at grafted sites and STSG donor sites (DS). Literature supports the use of ablative fractional CO2 laser (FLSR) to treat HTS, however many trials lack control sites and tissue-level examinations. Given the widespread adoption of FLSR for HTS, delegation of non-treated scar sites for the sake of RCT is troubling for many clinicians. We trialed using STSG DS scars for randomization rather than withholding FLSR from HTS at grafted sites.

**Methods:**

Patients (n=25) were treated for DS scar with FLSR. DS scars were randomized and treated with either 5 FLSR treatments, follow-ups, and standard of care (SOC) or SOC only. Prior to treatment, DS skin and normal skin (NS) were evaluated for trans-epidermal water loss (TEWL), melanin index (MI), elasticity, and erythema. Serial biopsies were analyzed for epidermal thickness, rete ridge ratio (RRR), and papillary dermal cellularity. All sites, including a separate STSG scar site, were evaluated using the patient and observer scar assessment scale (POSAS) and Vancouver Scar Scale (VSS).

**Results:**

Prior to treatment, DS skin had increased TEWL (10.5±0.8 vs 8.3±0.5 g/m2h, n=18; p=0.03), decreased RRR (1.1±0.0 vs 1.3±0.1, n=16; p=0.0001), and increased cellularity (8.8±0.9% vs 4.9 ± 0.6%, n=17; p=0.0014) compared to NS. DS skin and NS were not different in MI (p=0.07), erythema (p=0.77), elasticity (p=0.06), or epidermal thickness (p=0.32).

Over time, control site DS and laser-treated DS were not different in TEWL (p=0.92), elasticity (p=0.45), erythema (p=0.99), RRR (0.97), cellularity (0.99), MI, epidermal thickness, POSAS-O score, POSAS-P score, or VSS (p>0.99).

Over time, burn scar had increased skin elasticity (172.0±15.5 vs 78.5±13.2 N/m, n=17; p=0.0065). Burn scar did change in TEWL (p=0.53), MI (p=0.24), erythema (p=0.99), POSAS-O score (p=0.78), POSAS-P score (p=0.13), or VSS (p>0.99) over time.

**Conclusions:**

NS and DS skin possess inherent physiological differences, though not to the degree of STSG HTS vs. NS. FLSR may not alter the rate of maturation and remodeling of DS scar compared to current SOC. While improvement in scar assessment was observed in laser-treated STSG HTS, no specific control for these sites was analyzed. Due to differences in pathophysiology of HTS formation at STSG sites and DS, DS may not be an adequate substitute for STSG HTS when designing RCTs to evaluate the effect of FLSR.

**Applicability of Research to Practice:**

Prior studies evaluating the use FLSR consist of low-powered clinical trials or case studies without control sites or tissue level examinations, prompting the design of a RCT in DS scars. However, this scar type may not be suitable for this study design. Future work should extend to extra-cellular matrix morphology and transcriptomics of donor site and burn scar healing to better understand the effects of laser treatment.

